# Efficient design of meganucleases using a machine learning approach

**DOI:** 10.1186/1471-2105-15-191

**Published:** 2014-06-17

**Authors:** Mikhail Zaslavskiy, Claudia Bertonati, Philippe Duchateau, Aymeric Duclert, George H Silva

**Affiliations:** 1 Research and Development department, Cellectis, 8 rue de la Croix Jarry, Paris 75013, France

## Abstract

**Background:**

Meganucleases are important tools for genome engineering, providing an efficient way to generate DNA double-strand breaks at specific loci of interest. Numerous experimental efforts, ranging from *in vivo* selection to in silico modeling, have been made to re-engineer meganucleases to target relevant DNA sequences.

**Results:**

Here we present a novel in silico method for designing custom meganucleases that is based on the use of a machine learning approach. We compared it with existing in silico physical models and high-throughput experimental screening. The machine learning model was used to successfully predict active meganucleases for 53 new DNA targets.

**Conclusions:**

This new method shows competitive performance compared with state-of-the-art in silico physical models, with up to a fourfold increase in terms of the design success rate. Compared to experimental high-throughput screening methods, it reduces the number of screening experiments needed by a factor of more than 100 without affecting final performance.

## Background

Genome engineering (GE) focuses on the modification of genomes in living organisms at specific loci of interest. Examples of such modifications include insertion of a new gene into the genome, inactivation of existing genes via disruption of their sequences and replacement of a malfunctioning gene with a corrected version. GE has demonstrated its utility in basic research as well as in many industrial applications, for instance in agriculture and therapeutics [1-3]. One approach to GE is based on the use of sequence-specific nucleases to trigger DNA modifications by generating DNA double-strand breaks at the locus of interest. Currently, the four most frequently used tools in GE to generate targeted DNA cleavage are transcription activator-like effector nucleases (TALEN^a^) [4], CRISPR nuclease complexes [5,6], zinc-finger nucleases (ZFN) [4,7,8] and meganucleases (MN) [9,10]. These proteins have different properties (size, origin etc.), making it possible to match them with specific applications [3,11].

**Figure 1:**
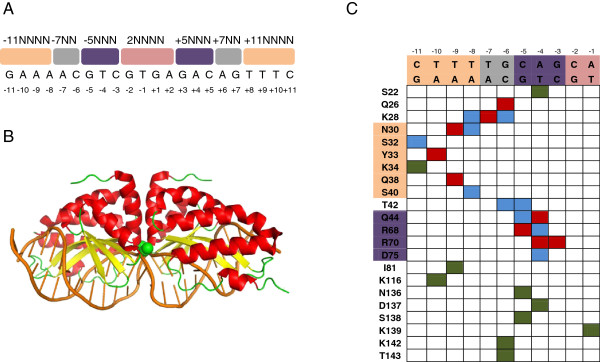
**I-CreI/DNA binding interface. (A)** Natural I-CreI target site with all positions indexed with respect to the center of the site from -11 to 11. -11NNNN and -5NNN are the reverse-complements of 11N4 and 5N3. **(B)** 3D structure of the I-CreI/DNA complex (PDB code: 1g9y). **(C)** I-CreI/DNA interaction map. Columns correspond to position on the DNA, rows correspond to positions of protein residues. Colors in the table are used to describe the nature of interaction between residues and nucleotides: dark green – backbone interactions, blue – water mediated, red – base specific. Residues N30-S40 and Q44-D75 are clustered together to indicate that they contact separate regions 11N4 and 5N3 on the DNA target.

Meganucleases are naturally occurring endonucleases characterized by large recognition sites (12 bp or more), which are almost unique in most genomes. The large recognition sites makes MNs perfect tools for GE, but unfortunately the number of naturally occurring MNs is quite limited and is not nearly sufficient to cover all potentially interesting loci. Therefore there was a strong need for a method that would allow us to redesign existing MNs to cut new DNA sequences. Existing redesign techniques include the creation of fusion chimeras from existing MN domains [12-14] and alteration of MN specificity via direct mutation of protein residues in the DNA binding scaffold [15-25]. One of the most used starting scaffolds for the design of new artificial MNs is I-CreI, a member of the LAGLIDADG family, the largest of five known families of MNs [26]. I-CreI is a homodimeric endonuclease cutting a 22 bp pseudopalindromic target (Figure 1A) with at least 25 known structures in the RCSB PDB [27] showing an alpha-beta(2)-alpha-beta(2)-alpha fold [28] (see Figure 1B). Figure 1C illustrates which residues participate in the binding of DNA [29]. In each monomer, residues R70 make direct contacts with base ±3 and ±4, Q44 with base ±4, and R68 with base ±5, while Q26 and K28 make direct contacts with bases ±6 and ±7s respectively. Positions ±10 make direct contacts with residue Y33 and ±9 with Q38 and N30. The base pairs at positions ±1 and ±2 (2N4 region) do not make direct contacts with any of the protein residues. Based on this interaction map, we previously defined four regions on the I-CreI target: the central 2N4 part of the recognition site (positions -2:2), where I-CreI cuts the DNA and three regions ±11N4, ±7N2, ±5N3 which constitute the binding sites [15-17].

Much effort has been made in the past to engineer I-CreI [15-21]. We formerly reported a successful combinatorial approach relying on the division of the I-CreI/DNA interface into separate clusters of amino acids with different DNA recognition regions. Namely, the fact that there is no intersection between groups of residues binding the 5N3 and 11N4 regions (see Figure 1C) makes it possible to screen for proteins binding target variants in the 5N3 and 11N4 regions independently and then combine the mutations from these two protein sets to cut a hybrid 11N4-5N3 target [15-17]. While conceptually the combination of different clusters allows efficient MN design, one still needs to screen hundreds of molecules to find the optimal MN. In [16], an assay based on single-strand annealing (SSA) was developed to perform such high-throughput screening as a routine assay. Another example of a semi-rational experimental approach is based on the sequential design of I-OnuI derived MNs [22]. Here, we present a new method for MN engineering that significantly reduces the number of molecules screened without reducing the final success rate.

Several studies have described the possibility of engineering MNs using atomistic molecular modeling software such as FoldX [19] and Rosetta [18,20,21]. These software packages build optimal MN structures by minimizing the binding energy of protein-DNA complexes. Most reported studies have limited the use of these packages mainly to the prediction of MNs for single base substitutions, but in [18] the authors reported successful MN prediction on targets with up to three base substitutions.

Diverse applications of machine learning (ML) approaches [30-36] have positioned machine learning techniques as promising tools for solving complex biological problems. Some of these applications, such as how to predict transcription factors, bear many similarities with the design of MNs. A necessary condition for the application of a ML approach is the existence of data from which a ML algorithm can learn a model. Over the years, we have gathered data on the cleavage activity of several hundreds of thousands of MN-DNA target pairs, which we used to train a machine learning model.

In this manuscript we present a new efficient method of MN design based on machine learning techniques. This strategy presents the advantage of a competitive success rate compared to that of experimental combinatorial high-throughput screening, while significantly reducing the number of screened molecules by several orders of magnitude and at the same time significantly outperforming alternative in silico models such as Rosetta and FoldX. The experimental validation of the ML approach lead to the successful design of MNs for 53 new DNA targets.

## Results

### Cross–validation experiments

The principal dataset used to train/cross-validate the machine learning model and to compare the performance of various in silico models consisted of 251 pseudopalindromic (reduction of non-palindromic targets to pseudopalindromic is described in [17]) 22 bp DNA targets screened according to a combinatorial process (see “Materials & methods”) giving in total 293k protein-DNA pairs of known activity (each target had at least one active protein).

In the first series of experiments we studied how in silico methods (ML approach and physical models) performed on the combinatorial dataset by doing cross-validation experiments (a detailed description of cross-validation scheme is given in Additional file 1: Figure S1). To assess the quality of model predictions, we computed several performance scores for each target: AUC score, Top10 score, and %Top10. Finally, the average value of each score over all targets from the test set was used as a global performance measure of in silico models (hereinafter we will use the simplified notations AUC, Top10, and %Top10 to denote the overall average values of each score).

**Figure 2:**
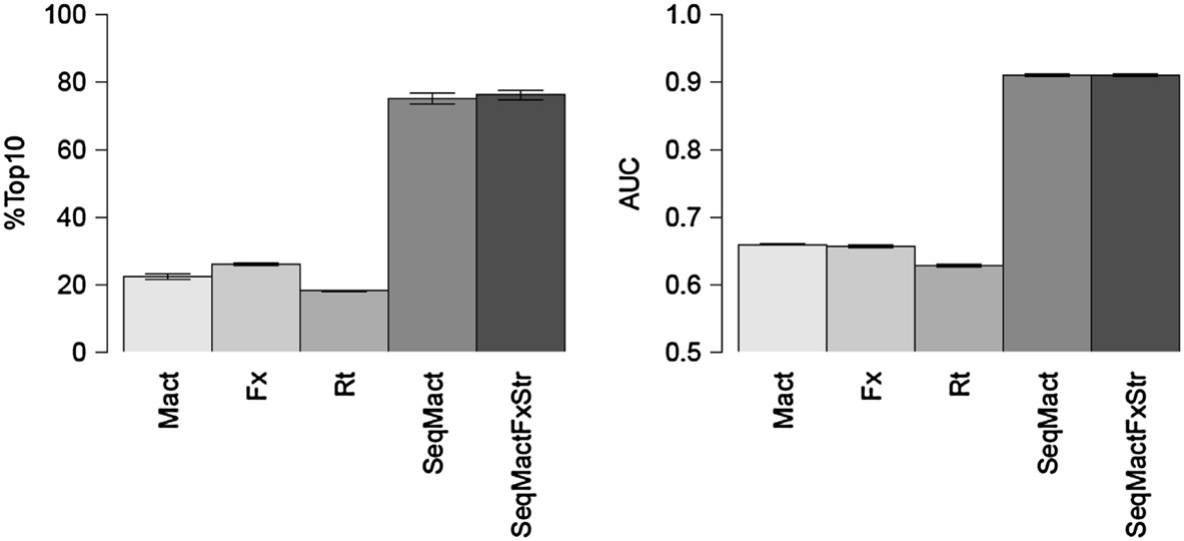
**Cross-validation performance of various in silico methods. (Left)** %Top10 — percentage of targets with at least one positive molecule in Top10 ranked, **(Right)** AUC – AUC score (see Material and Methods) Mact - predictions made on the basis of module cleavage activities, Fx — FoldX score, Rt — Rosetta score, SeqMact — protein/target sequences + module cleavage activities, SeqMactFxStr — all features combined (sequences + module cleavage activities + FoldX scores and interactions). Error bars are estimated from 30 independent cross-validation experiments.

Figure 2 presents the cross-validation performance scores (Top10% and AUC) for the following models: FoldX (Fx), Rosetta (Rt), Mact (machine learning model trained only on the cleavage activities of the p5N3 and p11N4 building modules), SeqMact (ML model trained on the cleavage activities of building modules plus target and protein sequences), SeqMactFxStr (ML model trained on all available features such as cleavage activities of building modules, target and protein sequences, FoldX scores and protein-DNA interaction maps). Values of Top10 score are given in Additional file 1: Figure S6. FoldX and Rosetta did not use the information available in the training set; they made their predictions by estimating the binding energy of the protein-DNA couple from a physical model. For a more detailed description of ML models, FoldX and Rosetta, see the corresponding sections of “Materials and methods”.

Overall Mact had an AUC score of around 0.66 and was able to predict at least one positive protein in the top 10 for about 20% of targets. Remarkably, the performance of the physical models (Fx and Rt) matched that of Mact. It is worth noting that Fx/Rt do not use information on module cleavage activities and can be used *ab initio* without any preliminary steps (i.e. screening of module libraries). However, when we added the information on protein and target sequences into the ML model (SeqMact), we obtained a significantly better success rate, predicting at least one positive mutant in the top 10 for about 80% of targets, with an AUC score of 0.9 (Additional file 1: Figure S13 shows the average ROC curve computed over test targets). The substantial difference in the success rates of Mact and SeqMact suggested that combining the best modules was not sufficient to get an active combined mutant; we also had to take into account protein and target sequence composition. Another important conclusion was that by learning sequence patterns specific to active and non-active proteins, we could obtain much better predictions than by exploiting a general physical model. Interestingly, when we combined SeqMact and Fx into a more general ML model (SeqMactFxStr) trained on sequence and structural features, we did not observe any improvement even though the two methods’ final protein rankings were not always the same.

**Figure 3:**
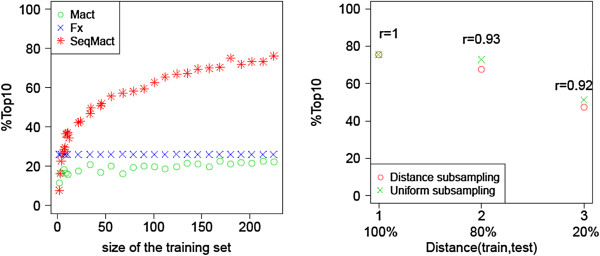
**Performance of ML model as a function of training set composition. (Left)** Performance of ML model as a function of the training set size (i.e. number of combinatorial libraries). Experimental setting are similar to those presented in Figure 2, where each point corresponds to the cross-validation performance when we use only a portion of the training data. **(Right)** Success rate as a function of the minimal distance between test and training targets (1, 2, 3) – distance in number of bases, (100%, 80%, 20%) – proportion of the training set which is kept after removal of targets which are too similar to targets in the test set. Distance subsampling – distance based selection of targets, Uniform subsampling – random selection of equivalent size training set; r gives the drop (ratio) in performance score due to the distance based selection of training targets.

Machine learning performance scores presented in Figure 2 correspond to the case when all available data in the training set were used to train the model (on average screening results on 226 = 0.9*251 targets). Figure 3 (Left) shows how Top10% (AUC and TopN are presented in Additional file 1: Figure S7) of Mact and SeqMact varies with the size of the training set, with only 10-20 targets (~10% of the actual traing set) SeqMact already matches the performance of FoldX and with 60 targets, it almost triples the performance. To make sure that SeqMact generalizes well on targets which are significantly different from what we already have in the training set, we repeated the previous experiment but this time instead of random subsampling of targets from the training set, we kept only those that had at least 2 and 3 base pair difference with all test targets (1 bp difference is the default situation where we use the entire training split). Figure 3 (Right) shows how the performance varied with the minimal allowed distance between training and test sets, we observe a drop in performance when the minimal distance (red circles) is increased. However the decrease is almost identical when we randomly sample an equivalent number of targets meaning that the model predicts well on significantly different targets and the observed drop in performance is due to the reduced size of the training set. Additional file 1: Figure S8 presents the distribution of all possible MN targets with respect to their distance from our training set. A majority of targets were at most 3 nucleotides distant from the training set, indicating that we could safely apply our method to almost all potential MN targets.

Another important question is how many molecules we needed to screen in order to have at least one positive mutant. Additional file 1: Figure S9 shows how the success rate of in silico approaches varied with the size of the screening pool. Ten molecules seemed to provide a good compromise between the number of molecules tested and the corresponding success rate; six molecules were enough to have on average one active molecule and at least one active molecule for more than 50% of the targets tested.

### Key features in machine learning model

In this section we address why SeqMact was more efficient than Mact. As described in “Cross-validation performance scores”, SeqMact can be seen as an extended version of Mact with additional features describing protein and target sequences. To determine whether any particular group of features contributed most to the performance boost, we tested alternative versions of SeqMact trained on several subgroups of features:

• SM-5 (SM-11 respectively): features encoding the p5N3 (p11N4 respectively) part of proteins and DNA sequences; no interactions between features; the relative performance of this model with respect to Mact reflected the importance of the p5N3 (p11N4 respectively) part of the sequences;

• SM-5_11: union of features from the two previous models; no interactions between features; the relative performance of this model with respect to SM-5 and SM-11 showed whether the combination of both parts (p5N3 and p11N4) improved performance with respect to the individual use of each part;

• SM-M2M: all features from the SM-5_11 model plus interactions between features encoding protein sequences; the relative performance of this model with respect to SM-5_11 reflected the impact of simultaneous protein mutations, i.e. whether there were any combinations of protein mutations that were harmful (or on the contrary favorable) to protein activity on any target (perhaps due to some folding or expression problems).

• SM-M2T: all features from the SM-5_11 model plus interactions between features encoding protein sequences and features encoding target sequences; this reflected the importance of dependencies between protein and target sequences, i.e. whether there were any preferences between target and protein sequences in addition to the already known cleavage activities of the p5N3 and p11N4 modules on the corresponding targets;

• SeqMact: union of all features used in previous models; the relative performance of SeqMact and SM-5_11 reflected the impact of 2nd order interactions between features encoding protein and target sequences, while the relative performance of SeqMact, SM-M2T and SM-M2M could tell us if there was any particular group of 2nd order interactions (M2M or M2T) contributing most to the performance boost.

In addition to the M2M versus M2T split, we also split 2nd order interaction features into features encoding dependencies within the same ‘module’ (interactions between features encoding the p5N3 part of the protein-DNA interface plus interactions between features encoding the p11N4 part) - SM-Intra, and 2nd order interaction features encoding dependencies between the p11N4 and p5N3 parts (i.e. cross-talk between combinatorial modules) - SM-Cross.

**Figure 4:**
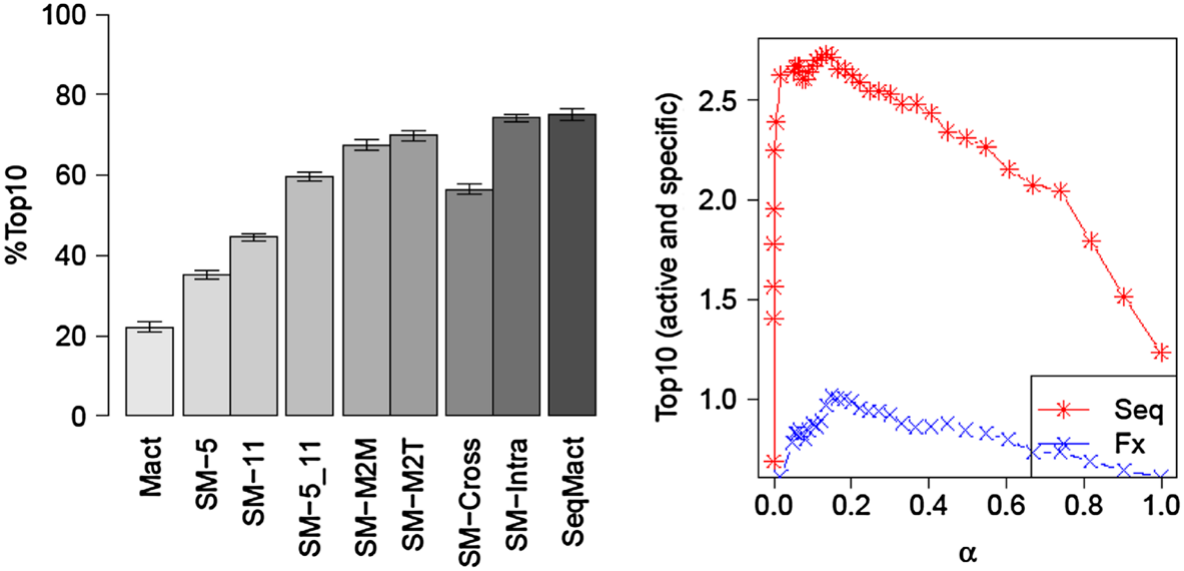
**Cross-validation performance of ML model as a function of interaction features. (Left)** %Top10 — percentage of targets with at least one positive molecule in Top10 ranked. Description of various groups of features (SM-5, SM-11, SM-5_11, SM-M2M, SM-M2T, SM-Cross, SM-Intra and SeqMact) are given in the text. Error bars are estimated from 30 independent cross-validation experiments. **(Right)** Prediction of active mutants at least as specific as the wild type I-CreI. Top10 — avg. number of active proteins at least as specific as I-CreI in top10 ranked molecules, α — trade-off parameter between predicted specificity and activity of candidate proteins. Seq – machine learning model trained on protein/target sequences, Fx – FoldX score.

The cross-validation performance of the alternative SeqMact versions is presented in Figure 4 (Left; Top10%, AUC and Top10 are given in Additional file 1: Figure S10). Overall, the use of information on protein sequences tripled the average number of active proteins in the top 10 predicted and the number of targets with at least one positive protein in the top 10 (SM-5_11 versus Mact). SM-5_11 basically learned which protein mutations were bad and which were good independently of the target sequence, i.e. non-specific activity patterns. The performance of SM-5_11 could be further enhanced by adding features describing simultaneous protein mutations (SM-M2M versus SM-5_11). SM-M2M learned only non-specific activity patterns, but these patterns were more sophisticated: now the model also learned whether there were any particular combinations of protein mutations that were good or bad for overall performance. When we added target-specific interactions (SM-M2T), we obtained an even higher performance boost. Finally, when we combined all features (SeqMact), the result was a model that outperformed both the SM-M2T and SM-M2M models, meaning that both types of interaction were important in the final model.

The relative performance of the SM-Cross and SM-Intra models suggests that the major performance boost obtained after adding interaction features came from the features describing dependencies within the same modules.

Examples of features with the strongest positive and negative impact in the model are given in Additional file 1: Table S1. The negative impact of the 44F mutation could be probably explained by the enhanced rigidity of the second beta-strand due to the contemporaneous presence of the wild type 43F; on the contrary the mutation 32K could have triggered additional non-specific DNA interactions. Electrostatic repulsion between 44R and 77R may have been the cause of the negative effect of the simultaneous presence of these two mutations in the protein (Additional file 1: Figure S12 illustrates the spatial proximity of 44R and 77R leading to an important interaction between them). Most of the features describing protein-DNA interactions corresponded to contacting positions, providing additional insights on which residues should or should not be used to target particular nucleotides.

### Activity versus specificity trade-off

Cleavage activity on the target of interest is not the only parameter we would like to control in MN, another important characteristic is its specificity. Since the prediction step was fast using the ML model compared to physical models, we could predict the activity of all candidate mutants on all possible targets in a matter of seconds and pick the mutant with the most specific profile or the mutant with the best trade-off between its predicted activity and specificity.

To assay experimentally a protein on all possible targets would require a tremendous amount of resources, so to validate our approach we used a set of 2,576 proteins mutated at positions contacting the 5N3 region and screened them on all 64 possible 5N3 targets (each mutant being active on at least one target). Screening all mutants on all targets enabled us to compute the specificity of all mutants in the same context (that of 5N3 targets). Similarly to [20], we computed the specificity of mutant pi on target tj as the ratio of the activity of pi on tj over the total activity of pi on all targets.

Rather than simply predicting the most active mutant, we were able to alter our criteria to favor the prediction of active mutants that were at least as specific as the wild type I-CreI on the same set of 64 5N3 targets (other activity/specificity trade-offs are possible as well). To predict mutants that were active and specific at the same time, we first predicted the activity of the candidate mutants on all 64 5N3 targets; we then computed the predicted specificity as the ratio of mutant activity on a given target of interest over the total activity on all 64 targets. Finally, we ranked all candidate mutants according to the following score combining predicted activity and predicted specificity:

$$R_{\alpha }=\mathrm{\alpha A}+\left(1-\alpha \right)S$$

where A and S are predicted activity and specificity, respectively.Figure 4 (Right) shows how the number of active mutants at least as specific as I-CreI in the top 10 varied as a function of α. When we ranked the candidate mutants according to their predicted activity, we obtained only a few specific mutants (α = 0), but when we output the most specific mutants we obtained very few active proteins. The results presented in Figure 4 were natural in the sense that if we wanted to predict not only active but also specific mutants we had to use a combined score representing a trade-off between the predicted activity and specificity of candidate mutants.

### De novo experiments: designing new custom MNs

In the second series of experiments, we tested the ability of our ML model to predict MNs on completely new DNA targets. The experiments were done on two groups of targets, a first group sampled from the extended target space (ETS) (38 targets), and a second group (39 targets) sampled from the restricted target space (RTS) (a subset of ETS) (see Additional file 1: Table S2 for the exact list of targets tested). The ETS is defined by a set of constraints on the sequences of 2N4 and 7N2 regions and the existence of active p5N3 and p11N4 building modules, it corresponds roughly to one target every 250 base pairs. These constraints were necessary to ensure a good probability that the combinatorial approach would find an active MN [15,37]. The RTS was defined as a subset of the ETS with additional constraints on the set of 2N4 sequences (only the most favorable 2N4 were allowed [38]), and 11N4-5N3 combinations (with the top 20% highest ML prediction scores), it corresponds to an average frequency of one target every 5.5kbp.

**Figure 5:**
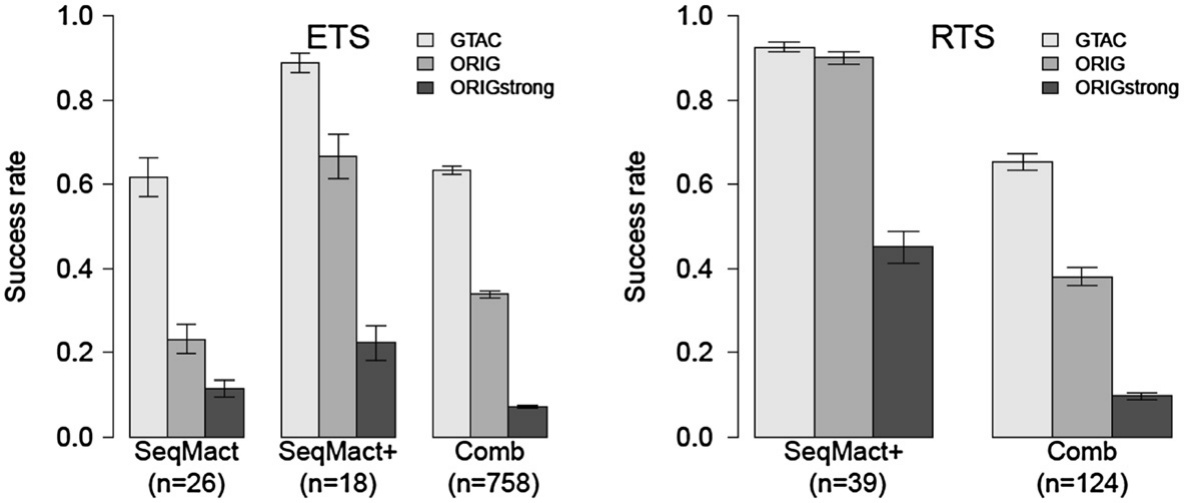
**Success rate of meganuclease design methods. (Left)** Experimental results on targets sampled from ETS (extended target space). **(Right)** Experimental results on targets sampled from RTS (restricted target space). SeqMact - machine learning predictions, SeqMact + — machine learning predictions with additional I132V mutation, Comb — combinatorial libraries. GTAC — proportion of GTAC target variants with at least one positive mutant, ORIG — proportion of original (sampled) targets with at least one positive mutant, ORIGstrong — proportion of original (sampled) targets with at least one highly active mutant (normalized cleavage activity score above 0.8).

For each target we predicted and tested 6 proteins (on average). Since the impact of 2N4 regions was independent of other regions and was not taken into account in the ML model, predicted mutants were also tested on variants of sampled targets with their 2N4 regions substituted by a GTAC sequence (the 2N4 sequence of the palindromic target derived from the left part of I-CreI wild type target and one of the most favorable 2N4 sequences [39]). Tests on GTAC target variants enabled us to see the success rate of the ML model regarding the quality of prediction of the optimal MN binding interface independent of any influence of the 2N4 region.In the first series of experiments, 156 MNs were predicted for 26 DNA targets sampled from the ETS. Figure 5 (Left) SeqMact presents the success rate (i.e. proportion of targets with at least one active MN) of ML predictions on the first group of targets (ETS). ORIG and GTAC denote the success rate over the original sampled targets and their GTAC variants, respectively. We also report the proportion of ORIG targets with at least one strongly active MN: ORIGstrong (cleavage activity score above 0.8). Overall, 62% (16/26) of GTAC targets and 23% (6/26) of ORIG targets were cut. Among the six ORIG targets cut, three had strongly active MNs.

It is known [40] that additional mutations such as I132V can help boost the activity of MNs. In the second series of experiments 72 MNs were predicted for another 18 targets sampled from the ETS, and this time all predicted MNs were synthesized with the additional mutation I132V. Figure 5 (Left) SeqMact + shows the success rate of ML predictions with additional I132V mutations. Although small sample sizes did not enable us to say that there was a statistically significant difference between success rates with and without the additional I132V mutation, the proportion of successfully cut GTAC targets went up to 83%, targets with original 2N4 up to 58%, and strongly cut original targets up to 16%.As a comparative reference, we report the success rate of the combinatorial process (Comb, high-throughput screening of about 1,160 proteins per target) on a much larger body of 758 targets also sampled from the ETS (see Figure 5 (Left) Comb). Overall, using ML predictions provided us with a success rate that was competitive in relation to that of high-throughput screening, with 200 times fewer molecules tested.

In the last series of experiments, we hypothesized that we could still improve the success rate of ML predictions by choosing targets that would be expected to be the easiest to cut [39]. To this end, 39 targets were thus randomly sampled from the more stringent RTS and assayed both with the original 2N4 and GTAC (Figure 5 (Right)). Indeed, the success rate on GTAC targets and targets with the original 2N4 went up to 92% and 90% respectively (42% strongly cut for original 2N4). The success rate of Comb on the RTS was the same as on the ETS.

## Discussion

Apart from a significant boost in performance, relatively low performance of Mact with respect to SeqMact models provided additional evidence to the hypothesis of non-negligible interdependency between p11N4 and p5N3 protein domains [15]. Furthermore the performance of alternative ML models trained on different subsets of features (SM-Cross and SM-Intra) suggested that the principal gain in performance was coming from a better selection of starting p11N4 and p5N3 modules that keep their activity profile when combined with each other, therefore providing a basis for the desirable factorization of the binding interface.

There are several explanations for the difference in performance between the ML approach and physical models. First, physical models rely on the computation of binding energy as the only important factor to predict active MNs. However in reality there are other factors such as protein expression and cleavage activity that can greatly influence the final activity of MNs. With the ML approach we directly model the final outcome, which may be much harder to handle with a physical model. Second, existing physical models use many approximations and simplifications that significantly reduce computation time, but which may have a negative impact on precision. The majority of the computational studies predicting DNA binding affinity for MNs have used a very conservative approach, only one base has been allowed to change. In [18] a triple base change was reported. In [20] many possible reasons for the difficulty of generating new in silico MNs are clearly discussed.

Whereas the main in silico experiments with physical models were carried out using only one structure, we also studied the possibility of using more than one structure to build our models. On 20% of the original combinatorial dataset we tested the performance of the method using three different structures (1g9b, 2vbj and 2xe0). No detectable improvements could be reported (data not shown), suggesting that a much wider structural coverage of potential MN/DNA target pairs is necessary to improve the success rate of physical models in large scale experiments.

Concerning the application of ML approaches to the design of new DNA-binding proteins, active learning methods [41] (instead of random sampling) as a more efficient way to collect the training data is an interesting direction for future research. Such a method would be especially useful when starting data collection for a new binding scaffold since it would help to choose the most informative examples for sampling and to reach faster the optimal success rate.

## Conclusions

In this study we address the application of a machine learning approach to the design of meganucleases cutting specific DNA targets. Our results are very promising in that the ML model significantly outperformed state-of-the-art in silico models such as FoldX and Rosetta. In addition, our method had success rates competing with those of combinatorial high-throughput screening, while reducing the number of molecules screened by several orders of magnitude. In experimental validation, the ML model successfully predicted active MNs for 53 new DNA targets. The boost in performance brought by the ML model comes with a price, as one needs a training set to learn the model, meaning it cannot be used completely *ab initio*. However, if a training set already exists, or if one is prepared to invest in building a new one, this could be considered a very interesting alternative to existing methods. In the case of MNs, experimental results on 20 DNA targets were already enough to train a machine learning model that outperformed existing in silico models.

## Methods

### Combinatorial process

A detailed description of the combinatorial process can be found in [17]. Here, we give a brief description of the procedure according to which the dataset of combinatorial libraries was generated. The goal of the combinatorial process was to find MNs that cut a given 22 bp DNA target T. We consider the case of palindromic target T (the case of a non-palindromic target can easily be reduced to the palindromic case by designing a separate meganuclease for the left and right parts of T; see [17]). The preliminary step in the combinatorial process involved creating module libraries of mutants cutting all possible 5N3 targets (64 targets) and all possible 11N4 targets (256 targets). Module libraries were created by degenerating a pool of I-CreI mutants at positions Q44, R68, R70, D75, I77 (hereinafter denoted by p5N3) contacting 5N3 regions and positions N30, S32, Y33, Q38, S40 (hereinafter denoted by p11N4) contacting 11N4 regions with further screening on all possible 5N3 (respectively 11N4) targets. This preliminary step needed to be done only once for all future targets. Since the sets of I-CreI interface residues contacting 5N3 and 11N4 regions were disjoint, a MN combining mutations of MNs cutting the 5N3 and 11N4 targets had a good chance of cutting the combined 11N4-5N3 target. In practice, testing a single combined MN rarely leads to success, and in order to have a good probability of finding an active MN up to 40 by 40 combinations (i.e. 1,600 combinations of 40 p5N3 MNs and 40 p11N4 MNs) have to be tested in high-throughput screening.

### Dataset of combinatorial libraries

When applying the combinatorial process to find active MNs for a given palindromic target of interest T, we started from the selection of MN modules targeting the 5N3 and 11N4 part of T, which were later used in the PCR assembly process (see “Combinatorial process”) to generate a number of final MNs p1,…,pK where pi is a combination of p5N3 mutations of a random p5N3 module and p11N4 mutations of a random p11N4 module. After screening candidate mutants p1,…,pK on the target of interest T, only positive mutants (if any) were sequenced. Although due to the random nature of the PCR assembly process we could not guarantee that all possible combinations of starting modules were present among the K candidate mutants, we could make the number of untested mutants quite low by oversampling (in our lab we sampled triple the theoretical diversity which should cover about 95% of all possible combinations). It was therefore natural to assume that 11N4-5N3 combinations, which are not found among positive sequences, were negative.

The dataset used in our ML computations consisted of 251 palindromic targets, all with GTAC (2N4 sequences of the palindromic target derived from the left part of I-CreI wild type target [39]) at their 2N4 region, screened according to the combinatorial process. This set was used to train/cross-validate the machine learning model and to compare the “cross-validation performance” of various in silico models. The average size of the combinatorial pools was 1,160 mutants. 26 targets had associated combinatorial pools with less than 500 mutants and 10 targets had pools of 2,000 or more mutants. The average number of positive mutants per target was 15, and 27 targets had only one positive mutant (all targets had at least one positive mutant). 19 targets had more than 40 associated positive mutants. Since only positive mutants were sequenced at the end of the combinatorial process, we could only be sure about the sequences of active proteins. The rest of the combinatorial pools are likely to have been negative and are considered, but not guaranteed, as such.

### 5N3 cross-validation set

The set of p5N3 mutants used to evaluate the “activity versus specificity trade-off” (see Section 3.3) consisted of 2,576 mutants tested on all 64 possible 5N3 DNA targets. 11,013 out of 164,864 (=64*2,576) protein-target pairs (6.6%) were positive, giving on average 172 positive mutants per target and 4 positive targets per mutant.

### Cross-validation performance scores

As explained above, the dataset consisted of a set of DNA targets t_i_ and associated candidate proteins p_ij_. We used in silico methods to compute an activity score A(t_i_,p_ij_) which was then used to rank candidate proteins p_i1_… p_iK_ for a given target t_i_. A perfect score would put all active proteins at the top of the list, but in practice we usually had a mix of positive and negative proteins, with the proportion of active proteins depending on the quality of the scoring function. To assess the quality of a ranking generated by a particular score, we used the following measures:

• %TopN – indicated whether there is at least one active molecule among the top N predicted; this score makes sense only when averaged over a set of targets; in this case it represents the percentage of targets where an in silico method was able to predict at least one positive in the top N (i.e. a successful experiment);

• TopN – in practice an in silico model is usually used to select a limited number of candidate molecules that will later be tested in real experiments. In this context, we used the number of active molecules among the top N predicted as a performance measure. In most of the figures, we used N = 10, but relative behavior of in silico methods was quite stable over various values of N (see “Cross-validation experiments” Additional file 1: Figure S1);

• AUC – area under the ROC (receiver operating characteristic) curve, a popular measure of global ranking quality [42]; one of the interpretations of this score is the probability that a random positive element (meganuclease in our case) will be ranked higher than a random negative element from the list of candidate proteins;

### ML model

The final machine learning model was an ensemble model combining GBM (gradient boosting machines with decision trees as basic learning models) [43] and LASSO (least absolute shrinkage and selection operator) models [44]. We used the gbm package [45] to train the GBM model and the glmnet package [44] to train the LASSO model. Classical SVM (hinge loss, L2 regularization) showed inferior performance, but rankSVM model (optimization of the AUC score) was competitive with LASSO and GBM. A variant of the GBM model based on the optimization of the AUC score lead to a slight improvement in terms of AUC, but had a rather negative impact on Top10 and %Top10. The final ensemble combination was restricted only to LASSO and GBM, addition of other models had no significant impact on the performance of the ensemble model. Parameters of ML models were systematically estimated from inner cross-validation loops on training folds (see Additional file 1: Figure S2) and then used to test the final model on the test fold. Additional file 1: Figure S3 shows the performance of these models separately and when combined together in an ensemble model. A detailed description of the algorithm used to build the ensemble model is given in Additional file 1: Figure S11.

GBM was trained on categorical features representing mutations at 11 key positions in the protein sequence, the DNA target sequence and cleavage activities of starting p5N3 and p11N4 building modules (a detailed description of the dataset is given in Additional file 1: Figure S4 “Categorical dataset”). To train the LASSO model, all categorical features were encoded using binary features (with each binary feature encoding a particular mutation of the protein sequence or nucleotides in the DNA sequence). In addition, products of all pairs of individual features with more than 200 non-zero components were added to the model to encode second-order interactions between features (detailed description of the datasets is given in Additional file 1: Figure S4 “Bin1” and “Bin2” dataset).

## Endnote

^a^TALEN™ is a trademark owned by Cellectis bioresearch.

## Competing interests

All authors are employees of Cellectis SA. A.D and P.D. have stocks or stock options of Cellectis SA.

## Authors’ contributions

MZ performed the data processing, built the machine learning model, participated in the design of the study and drafted the manuscript. CB carried out computational biology experiments, participated in the design of the study and drafted the manuscript. AD coordinated the data processing, participated in the design of the study and drafted the manuscripts. PD and GS conceived of the study, participated in its design and coordination and helped to draft the manuscript. All authors read and approved the final manuscript.

## Supplementary Material

Additional file 1: Figure S1 Cross-validation experiments. **Figure S2.** Performance of individual ML models in inner cross-validation loops as a function of model parameters. **Figure S3.** Relative performance of individual ML models and their ensemble combination. **Figure S4.** Dataset representations (features) used in various machine learning models. **Figure S5.** Examples of yeast experimental results with corresponding values of normalized cleavage activity score. **Figure S6.** Top10 cross-validation performance of various in silico methods. **Figure S7.** Performance of ML model as a function of the training set size (i.e. number of combinatorial libraries), experimental setting are similar to that presented in Figure 2, each point corresponds to the cross-validation performance when we use only a portion of the training data. **Figure S8.** Number of all potential meganuclease targets as a function of their distance to the training set. **Figure S9.** Success rate as a function of the number of molecules tested. (Left) Average number of active molecules in TopN predicted. (Right) Proportion of targets with at least one positive mutants in TopN predicted. Mact — predictions made on the basis of module cleavage activities, Fx — FoldX score, SeqMact — protein/target sequences + module cleavage activities. **Figure S10.** Cross-validation performance of ML model as a function of interaction features. (Left) AUC — AUC score, (Right) Top10 — avg. number of positives in top10 ranked molecules. **Figure S11.** Pseudo-code of the ensemble model estimation in the cross-validation experiments. **Figure S12.** Spatial positions ofamino-acids 32, 40, 44 and 77 in the protein-DNA binding complex. **Figure S13.** Average ROC curve computed from SeqMact model predictions. **Table S1.** Examples of features and feature interactions having positive and negative impact on the activity of meganucleases. **Table S2.** List of DNA targets tested in Section “De novo experiments”. ORIG– highest achieved activity on the corresponding target, GTAC –highest achieved activity on the GTAC target variant (2N4 substituted by GTAC). Click here for file
