# Supplementary material for: Efficient design of meganucleases using a machine learning approach
Source: BMC Bioinformatics. 2014 Jun 17;15:191. doi: 10.1186/1471-2105-15-191 (PMC4065607; doi:10.1186/1471-2105-15-191)
Supplement: Additional file 1: Figure S1 — Cross-validation experiments. Figure S2. Performance of individual ML models in inner cross-validation loops as a function of model parameters. Figure S3. Relative performance of individual ML models and their ensemble combination. Figure S4. Dataset representations (features) used in various machine learning models. Figure S5. Examples of yeast experimental results with corresponding values of normalized cleavage activity score. Figure S6. Top10 cross-validation performance of various in silico methods. Figure S7. Performance of ML model as a function of the training set size (i.e. number of combinatorial libraries), experimental setting are similar to that presented in Figure 2, each point corresponds to the cross-validation performance when we use only a portion of the training data. Figure S8. Number of all potential meganuclease targets as a function of their distance to the training set. Figure S9. Success rate as a function of the number of molecules tested. (Left) Average number of active molecules in TopN predicted. (Right) Proportion of targets with at least one positive mutants in TopN predicted. Mact — predictions made on the basis of module cleavage activities, Fx — FoldX score, SeqMact — protein/target sequences + module cleavage activities. Figure S10. Cross-validation performance of ML model as a function of interaction features. (Left) AUC — AUC score, (Right) Top10 — avg. number of positives in top10 ranked molecules. Figure S11. Pseudo-code of the ensemble model estimation in the cross-validation experiments. Figure S12. Spatial positions ofamino-acids 32, 40, 44 and 77 in the protein-DNA binding complex. Figure S13. Average ROC curve computed from SeqMact model predictions. Table S1. Examples of features and feature interactions having positive and negative impact on the activity of meganucleases. Table S2. List of DNA targets tested in Section “De novo experiments”. ORIG– highest achieved activity on the corresponding target, GTAC –highest achie [file 1471-2105-15-191-S1.docx]

# Supplementary Figures and Tables

**Cross-validation experiments (Figure 2):**

1. The set of protein-target pairs is randomly split into 10 disjoint parts (folds), the splits are made to ensure that all protein-target pairs with identical targets belong to the same fold.
2. Repeatedly, for each fold, we train machine learning models (Mact, SeqMact, SeqMactFxStr) on the other 9 “training” folds and then use the trained model to make prediction on the fixed “test” fold. Model tuning (selection of optimal parameters) is made via internal cross-validation on the set of training folds; no information from the test fold is used. Once predictions are made, we use these values to rank candidate proteins for each target and we compute various performance scores to assess the quality of protein ranking.
3. The final performance score is computed as the average value of performance scores over all targets.

**Cross-validation experiments (Figure 3(Left)):**

The same steps were done as in the previous case, except that at step 2 instead of using all targets from training folds, we randomly sample a subset of targets (and corresponding protein-target pairs) and use only these targets to train machine learning models.

**Cross-validation experiments (Figure 3(Right)):**

The same steps were done as in Figure 3(Left), but instead of random sampling, we select training targets which are at least 1,2,3 bp distant from all test targets.

Supp. Figure 1. Cross-validation experiments.

|  | **AUC** | **Top10** | **%Top10** |
| --- | --- | --- | --- |
| **GBM** |  |  |  |
| **Lasso2** | **** | **** | **** |
| **RankSVM** |  |  |  |
| **SVM2** |  |  |  |

Supp. Figure 2. Performance of individual ML models in inner cross-validation loops as a function of model parameters.

|  | **AUC** | **Top10** | **%Top10** |
| --- | --- | --- | --- |
| **SVM** | .824±.001 | 1.226±.025 | .557±.011 |
| **SVM2** | .893±.001 | 1.813±.025 | .589±.01 |
| **RankSVM** | .909±.001 | 2.109±.025 | .726±.01 |
| **Lasso** | .861±.001 | 1.336±.023 | .597±.009 |
| **Lasso2** | .91±.001 | 2.096±.02 | .747±.008 |
| **GBM** | .903±.001 | 1.841±.017 | .713±.003 |
| **RankGBM** | .904±.001 | 1.785±.015 | .705±.003 |
| **Ensemble** | .915±.001 | 2.134±.011 | .757±.002 |

Supp. Figure 3. Relative performance of individual ML models and their ensemble combination. SVM – linear support vector machine model (hinge loss, L2 regularization) trained on the Bin1 representation (no feature interactions, see Supp.Figure 5), SVM2 – linear support vector machine model (hinge loss, L2 regularization) trained on the Bin2-representation (2nd order feature interactions), RankSVM – linear rank SVM model (direct optimization of the AUC score, L2 regularization) trained on the Bin2 representation, Lasso – lasso model (logistic loss function, L1 regularization) trained on the Bin1 representation, Lasso2 – lasso model (logistic loss function, L1 regularization) trained on the Bin2 representation, GBM – gradient boosting machine model (binomial aka logistic regression loss function) trained on the Cat representation, RankGBM – gradient boosting machine (direct optimization of the AUC score), Ensemble – ensemble combination of Lasso2 and GBM.

- SVM and SVM2 models were trained with liblinear package [http://www.csie.ntu.edu.tw/~cjlin/liblinear/]: regularization parameter C was estimated from the inner cross-validation loop on the training data.
- RankSVM was trained with liblinear-ranksvm package [http://www.csie.ntu.edu.tw/~cjlin/libsvmtools/]: regularization parameter C was estimated from the inner cross-validation loop on the training data.
- Lasso and Lasso2 were trained with glmnet package [http://www.jstatsoft.org/v33/i01/]: regularization parameter λ was estimated from the inner cross-validation loop on the training data.
- GBM and RankGBM were trained with gbm package [http://code.google.com/p/gradientboostedmodels/], shrinkage, interaction depth and number of trees were estimated from the inner cross-validation loop.
- Ensemble model was trained as a linear combination of GBM and Lasso2 models, coefficients of the linear combination were estimated from the inner cross-validation loop used to optimize GBM and Lasso2 parameters.

In addition to linear SVM models, polynomial and Gaussian kernels were tested as well (lasvm on the entire dataset and libsvm on a smaller subset of data) on B1 and B2 but did not show any improvement with respect to SVM2. Testing of linear SVM and Lasso on a binary dataset containing 3rd order feature interactions [only a subset of 3rd order interactions was added to make the training tractable] did not show any detachable improvement in performance with respect to B2. Direct optimization of the AUC score (RankSVM and RankGBM) had a positive effect on the AUC score, but was not always favorable in terms of Top10 and %Top10 scores. The final Ensemble model was constructed from only two baseline models (GBM and Lasso2), addition of other models did not lead to any improvement in performance.

**“Cat”** categorical dataset representation:

Each protein-target pair is described by the following vector of features

1. 14 categorical features (each taking 20 possible values ‘A’,’R’,’N’,..) describing protein mutations at positions (24, 28, 30, 32, 33, 38, 40, 44, 68, 70, 75, 77, 80, 139)
2. 9 categorical features (each taking 4 possible values ‘A’, ‘T’, ‘C’, ‘G’) describing DNA target composition at positions -11 -10 -9 -8 -7 -6 -5 -4 -3

3. One numerical feature (real value between 0 and 1) describing the activity of p5N3 module (only mutations at positions 24, 44,68,70,75,77,80, 139 are kept) on the corresponding 5N3 target (wild type ICre-I target where base pairs at positions -5 -4 -3 are replaced with base pairs at the corresponding positions from the target of interest)

4. One numerical feature (between 0 and 1) describing the activity of p11N4 module (only mutations at positions 28,30,32,33,38,40 are kept) on the corresponding 11N4 target (wild type ICre-I target where base pairs at positions -11 -10 -9 -8 are replaced with base pairs at the corresponding positions from the target of interest).

The dataset is represented by a 293114x25 matrix (293114 is the total number of protein-target pairs in the dataset) and a 293114 dimensional vector of outcomes (real value between 0 and 1). Each line of the matrix corresponds to a particular experimental result of a protein on a target. For example, if protein 24I28R30Q32H33Y38R40I44R68V70N75A77L80K139V composed of two modules 28R30Q32H33Y38R40I (p11N4) and 24I44R68V70N75A77L80K139V (p5N3) showed activity of 0.9 on target TACAACCCTGCATAGGGTTGTA, this experimental result would be recorded in the following form

| X1 | X2 | X3 | X4 | X5 | X6 | X7 | X8 | X9 | X10 | X11 | X12 | X13 | X14 | X15 | X16 | X17 | X18 | X19 | X20 | X21 | X22 | X23 | X24 | X25 | Y |
| --- | --- | --- | --- | --- | --- | --- | --- | --- | --- | --- | --- | --- | --- | --- | --- | --- | --- | --- | --- | --- | --- | --- | --- | --- | --- |
| I | R | Q | H | Y | R | I | R | V | N | A | L | K | V | A | C | A | A | C | C | C | T | C | 0.5 | 0.8 | 0.9 |

where positions X1-X14 encode protein mutations, positions X15-X23 encode DNA target compositions, X24 contains the activity of p11N4 module 28R30Q32H33Y38R40I on 11N4 target TACAACGTCGCATGACGTTGTA and X25 contains the activity of p5N3 module 24I44R68V70N75A77L80K139V on 5N3 target TAAAACCCTGCATAGGGTTTTA, Y contains the activity of the combined mutant on the target of interest.

**“Bin1”** binary dataset representation**:**

All categorical features in “Categorical representation” (columns X1-X25 in the example above) are replaced with groups of binary features representing particular mutations on specific positions i.e. one feature from the categorical dataset describing mutations at position 44 is replaced with up to 20 binary features 44A, 44R, 44N etc (we remove features describing mutations which never occur at the given position). Similarly one feature described a DNA target base pair is replaced with 4 binary features. In other words, each of X1-X14 is now represented by 20 binary columns encoding particular amino acids, and each of X15-X23 is now replaced with 4 binary columns encoding particular nucleotides.

The dataset is represented by a 2931114x226 matrix and a 293114 dimensional vector of outcomes (real value between 0 and 1).

**“Bin2”** 2^nd^ order binary representation describing 2^nd^ order feature interactions:

This dataset is an extended version of the previous one, where we add pairwise products between all features from the “Binary representation” (we keep only products with at least 200 non-zero elements, which provide a good compromise between model performance and model training time).

The dataset is represented by a 293114x6775 sparse matrix and a 293114 dimensional vector of outcomes (real value between 0 and 1).

|  |  |  |  |
| --- | --- | --- | --- |

Supp. Figure 4. Dataset representations (features) used in various machine learning models.


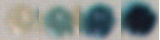


| 0.0 | 0.4 | 0.8 | 1.0 |
| --- | --- | --- | --- |

Supp. Figure 5. Examples of yeast experimental results with corresponding values of normalized cleavage activity score.

Supp. Figure 6. Top10 cross-validation performance of various in silico methods. Mact - predictions made on the basis of module cleavage activities, Fx — FoldX score, Rt — Rosetta score, SeqMact — protein/target sequences + module cleavage activities, SeqMactFxStr — all features combined (sequences + module cleavage activities + FoldX scores and interactions). Error bars are estimated from 30 independent cross-validation experiments.

###

Supp. Figure 7. Performance of ML model as a function of the training set size (i.e. number of combinatorial libraries), experimental setting are similar to that presented in Figure 2, each point corresponds to the cross-validation performance when we use only a portion of the training data. (Left) AUC – AUC score, (Right) Top10 — avg. number of positives in top10 ranked molecules. Mact - predictions made on the basis of module cleavage activities, Fx — FoldX score, SeqMact — protein/target sequences + module cleavage activities.

###

Supp. Figure 8. Number of all potential meganuclease targets as a function of their distance to the training set.

###

Supp.Figure 9. Success rate as a function of the number of molecules tested. (Left) Average number of active molecules in TopN predicted. (Right) Proportion of targets with at least one positive mutants in TopN predicted. Mact — predictions made on the basis of module cleavage activities, Fx — FoldX score, SeqMact — protein/target sequences + module cleavage activities.

###

###

Supp. Figure 10. Cross-validation performance of ML model as a function of interaction features. (Left) AUC — AUC score, (Right) Top10 — avg. number of positives in top10 ranked molecules. SM-5 – individual features describing 5N3 domains, SM-11 – individual features describing 11N4 domain, SM-5_11 - individual features from SM-5 and SM-11; SM-M2M —SM-5_11 features plus mutant to mutant interactions, SM-M2T — SM-5_11 features plus mutant to target interactions, SM-Cross — SM-5_11 features plus cross interactions between 5N3 and 11N4 regions,SM-Intra — SM-5_11 features plus intra interactions within 5N3 and 11N4 regions, SeqMact — all features are used. Error bars are estimated from 30 independent cross-validation experiments.

**Input:** The dataset of experimental results (feature matrix X and vector of outcomes Y)

1. Split lines of matrix X (and Y) into ten disjoint sets S1…S10 in such a way that experimental results corresponding to a particular target are placed in one set.
2. External cross validation: for each set Si do
   1. Generate a new dataset Xi and Yi by removing lines Si from the original dataset
   2. Split Xi (and Yi) into ten disjoint sets M0,M1…M10 (again experimental results on a particular target are placed in one subset)
   3. For each subset Mi, i=1..10
      1. build glmnet model on {M1…M10}\Mi for lambda=exp(-10:0)

*R>> m1=glmnet(Xi[-Mi,],Y[-Mi],lambda=exp(-10:0));*

- - 1. build gbm model on {M1…M10}\Mi

*R>>m2=gbm(Y~.,data=data.frame(Xi,Yi)[-Mi,], interaction.depth=idep,shrinkage=shr,n.trees=10000)*

For idp = {1,2,3,4,5} and sh = {1e-4,1e-3,1e-2}

- - 1. Compute model performance on subset Mi (AUC, Top10 or %Top10)
  1. Select the values of parameter lambda for glmnet and (shrinkage,interaction depth) for gbm which give the best average performance scores on test subsets Mi.
  2. Use optimal parameters to predict activity scores for subset M0 and build a linear model (ensemble model) on the top of glmnet and gbm predictions by using M0 subsample.
  3. Compute performance of the ensemble model on Si (AUC,Top10 or %Top10).

**Output:** Cross-validation performance scores.

Supp. Figure 11. Pseudo-code of the ensemble model estimation in the cross-validation experiments.


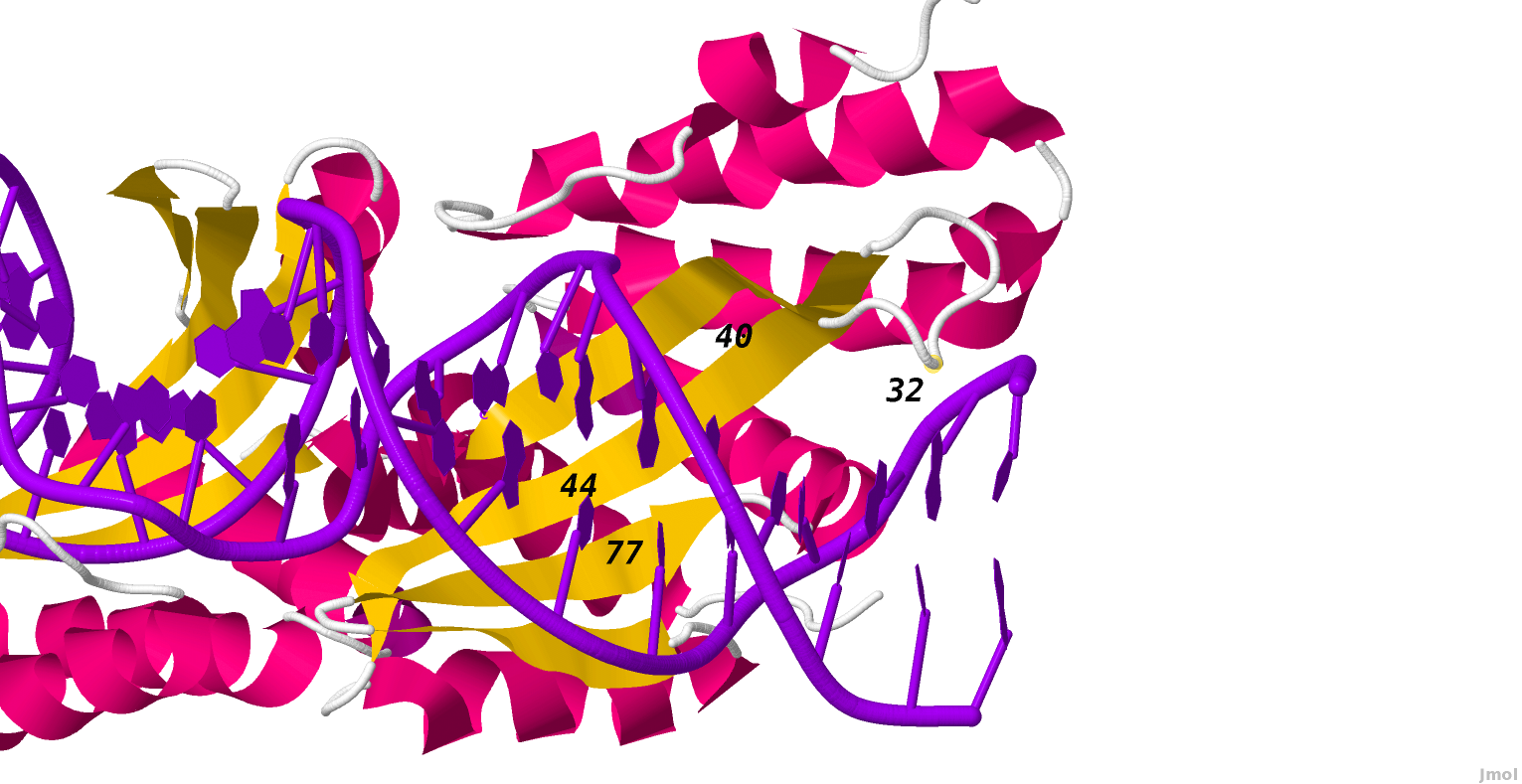


Supp. Figure 12. Spatial positions of amino-acids 32, 40, 44 and 77 in the protein-DNA binding complex.

Supp. Figure 13. Average ROC curve computed from SeqMact model predictions. Red points represent average values of true positive rates for a fixed value of false positive rate over test targets, error bars correspond to the standard deviation of computed true positive rates (not divided by square root of the number of test targets).

Supp. Table 1. Examples of features and feature interactions having positive and negative impact on the activity of meganucleases.

|  | Individual mutations | Interactions within protein sequence | Interactions between protein and target sequences |
| --- | --- | --- | --- |
| Negative impact | \| 44F \| \| --- \| \|  \| | \| 30Q 33H \|  \| \| --- \| --- \| \| 32D 40Q \|  \| \| 44R 77R \|  \| | \| 33R 10T \| \| --- \| \| 68E 6T \| \| 44R 4A \| \| 77L 4A \| |
|  |  |  |  |
| Positive impact | \| 32K \| \| --- \| \| 33M \| | \| 30K 33A \| \| --- \| \| 28R 38Y \| \| 33R 38G \| | \| 38R 9G \| \| --- \| \| 77K 7A \| \|  \| |

Supp. Table 2. List of DNA targets tested in Section "De novo experiments". ORIG– highest achieved activity on the corresponding target, GTAC –highest achieved activity on the GTAC target variant (2N4 substituted by GTAC).

| **DNA target sequence** | **ORIG** | **GTAC** |
| --- | --- | --- |
| TAAAACCCTCATAAGAGGGTTTTA | 0.66 | 0.90 |
| TAAAGCCACTTTAAAGTGGCTTTA | 0.00 | 0.00 |
| TAAGGATCATGTATATGATCCTTA | 0.72 | 0.85 |
| TAAGGATTCCGAACGGAATCCTTA | 0.00 | 0.33 |
| TAGACACGTCATAAGACGTGTCTA | 0.00 | 0.00 |
| TAGAGATCTCGTAAGAGATCTCTA | 0.95 | 0.96 |
| TAGGACTACCGAACGGTAGTCCTA | 0.00 | 0.60 |
| TATAACTATTGTATAATAGTTATA | 0.63 | 0.69 |
| TATAACTCTGGCAACAGAGTTATA | 0.54 | 0.84 |
| TCAATATAATGCAAATTATATTGA | 0.95 | 0.97 |
| TCAATCTTATGAACATAAGATTGA | 0.88 | 0.98 |
| TCAGACTGGCGTATGCCAGTCTGA | 0.63 | 0.87 |
| TCAGCACCACTTAAGTGGTGCTGA | 0.00 | 0.89 |
| TCCATATCAGGAACCTGATATGGA | 0.74 | 0.86 |
| TCCCTCCCCTGTGTAGGGGAGGGA | 0.31 | 0.68 |
| TCGACATTATGCTCATAATGTCGA | 1.00 | 1.00 |
| TCGACATTTCGTATGAAATGTCGA | 0.88 | 0.93 |
| TCGATACGACGTGCGTCGTATCGA | 1.00 | 1.00 |
| TCGATATCCCGTAAGGGATATCGA | 1.00 | 1.00 |
| TCGATCCCCCGTGCGGGGGATCGA | 1.00 | 1.00 |
| TCGATCTCCCGTAAGGGAGATCGA | 1.00 | 1.00 |
| TCGCACTCAGGCTCCTGAGTGCGA | 0.98 | 1.00 |
| TCGCTCTTATGTATATAAGAGCGA | 1.00 | 0.99 |
| TCGTCCCCACATAAGTGGGGACGA | 0.80 | 1.00 |
| TCTACACGATGTGAATCGTGTAGA | 0.50 | 0.43 |
| TCTAGCTGTCGCAAGACAGCTAGA | 0.92 | 1.00 |
| TCTATCCAAGGTGACTTGGATAGA | 0.59 | 0.84 |
| TCTCGCCCATTTAAATGGGCGAGA | 0.43 | 0.99 |
| TCTCTCCTTCGTGAGAAGGAGAGA | 1.00 | 1.00 |
| TCTGAATAACGCAAGTTATTCAGA | 0.18 | 0.76 |
| TCTGGACTATGTGTATAGTCCAGA | 0.80 | 0.92 |
| TGAAGACCTATTAATAGGTCTTCA | 0.00 | 0.88 |
| TGGAGACCTCGTGCGAGGTCTCCA | 1.00 | 1.00 |
| TGTAGCCCTCGTGCGAGGGCTACA | 1.00 | 1.00 |
| TGTATATGGAGCAATCCATATACA | 0.37 | 0.96 |
| TTACGCTCCCGCAAGGGAGCGTAA | 0.31 | 0.66 |
| TTATTATCCCGTATGGGATAATAA | 0.00 | 0.62 |
